# Center of pressure progression patterns during level walking in adolescents with idiopathic scoliosis

**DOI:** 10.1371/journal.pone.0212161

**Published:** 2019-04-22

**Authors:** Chia-Chi Gao, Jen-Suh Chern, Chun-Ju Chang, Po-Liang Lai, Chi-Wen Lung

**Affiliations:** 1 Department of Occupational Therapy and Graduate Institute of Behavioral Science, School of Medicine, Chang Gung University, Taoyuan, Taiwan; 2 Occupational Therapy in Rehabilitation Department, Far Eastern Memorial Hospital, New Taipei City, Taiwan; 3 Graduate Institute of Rehabilitation Counseling, National Taiwan Normal University, Taipei, Taiwan; 4 Master of Business Administration Program, Rotterdam School of Management, Erasmus University, Rotterdam, Netherlands; 5 Department of Orthopaedic Surgery, Chang Gung Memorial Hospital, Taoyuan, Taiwan; 6 College of Medicine, Chang Gung University, Taoyuan, Taiwan; 7 Department of Creative Product Design, Asia University, Taichung, Taiwan; Texas Scottish Rite Hospital for Children, UNITED STATES

## Abstract

The purpose of this study was to determine whether functional walking performance measured with Timed Up-and-Go (TUG) and center of pressure (CoP) progression pattern is different across adolescents with various curve severity of idiopathic scoliosis (IS). The CoP coordinates during a stance phase for self-paced level walking between adolescent with three different severities of IS (mild IS, moderate IS, and severe IS) and age-matched normal subjects were measured with foot pressure measurement. The average data of three trials were compared among groups with repeated measure analysis of variance. Results showed that the TUG was different between normal and AIS subjects, indicating use of TUG as a marker of functional walking performance in AIS is plausible but studies with larger sample size is needed before using TUG to identify AIS with different scoliosis severity. The results also showed that the CoP displacement, velocity and acceleration during a stance phase was different across groups, and with the most prominent deviations found in the moderate IS group. The medial-lateral shifting of the CoP trajectory at mid-foot regions in all IS groups deviated the most. A tendency of asymmetry in CoP progression pattern between feet in IS groups was also found. The deviation of the spine alignment in frontal plane could change the CoP progression patterns during level walking, suggesting the risk of the locomotors subjecting to abnormal loading during daily walking. Education and conservative interventions might be needed for preservation of medical outcome and prevention of back pain and/or musculoskeletal consequences later in the lives of AIS with and without surgical correction.

## Introduction

Level walking is the most frequent performed daily movement, which requires that the body endures more than our body weight repeatedly for each step we take. The biomechanical alignment of segments within the movement linkage during walking and accompanying sensory-perceptual-motion control mechanism make human walking both energy efficient and safe [1]. Changes in walking pattern expose our body to abnormal loading pattern and increase the risk of accumulated musculoskeletal injury to the segments between the spine and the foot [2]. Therefore, understanding the loading pattern during level walking is pivotal for prevention of musculoskeletal discomfort accumulated from daily walking.

Idiopathic scoliosis (IS) is the most common deformity of the spine in humans and forms in early life. The severity of IS, which is measured by Cobb’s angle, progresses with time in 15%~60% of this population, especially in patients diagnosed of IS in their adolescent ages (AIS) [3–4]. Changes of standing stability and gait characteristics during level walking in AIS population was documented by several researches [2,5–7] but with a moderate risk of research bias [8], which partially ruled out neuropathogenesis of AIS. The reason might be that the kinematic parameters used in the studies [7,9], including the step length, length of stance phase, cadence, range of motion at lower limb joins, acceleration at pelvis during loading response, and mid-stance and pre-swing sub-phases, were not valid and/or reliable enough in documenting the changes of gait in the AIS population.

As previously mentioned, the incidence of scoliosis severity progression is high, and surgery remains the best way to correct and stabilize the deformity. Bruyneel et al [9] found that kinematic parameters measured during side-stepping were not reliable gait parameters for AIS with various scoliosis severity; and Mahadens et al [10] reported the minimal changes of kinematic gait parameters at one year after surgery correction of scoliosis severity and that there was a trend of increasing center of pressure (CoP) medial-lateral displacement during level walking as a function of scoliosis severity. Therefore, it is reasonable to hypothesize that CoP might be valid and reliable functional markers for identification of subjects with various IS severity.

CoP is the location at which the instantaneous vector of the ground reaction force acts when the plantar surface contacts the ground. It is a two-dimensional position parameter and its location changes constantly along the time course during control upright posture stability and walking pattern. The amount of CoP sway, which is defined as the posture sway, is a well-accepted measure of standing stability and a representation of the intensity of neural commands for stability control from higher centers in the brain [11–12] On the other hand, the CoP under each of the two plantar surfaces is a unique measure that can provide insights for the loading patterns into the stance phase of a gait cycle while walking [13], which is not replaceable by kinematics parameters. During level walking, the plantar CoP pathway initiate at the hindfoot (initial contact) and terminates at the forefoot (toe off) synchronizing with sequential foot rockers, i.e. heel rocker, midfoot rocker, forefoot rocker and toe rocker, within the ankle-foot complex. Its progression within the plantar surface shows the loading pattern that is accepted by the foot and transmitted upwardly to the skeletal components above the foot, including the spine [11]. Changes of CoP progression might be caused by impaired neural control for gait [5] and/or biomechanical misalignment and inter-joint coordination of segments within the linkage system between spine and the plantar surface [12].

Back pain is a frequently reported physical discomforts for AIS. The severity of pain was rated no less than five points on visual analogue scale in more than 50% of this population. Furthermore, evidences clearly show that the severity of scoliosis is associated with the severity of pain [14–15]. Up to 73% AIS patients without underwent surgical correction reported at least one episode of back pain at initial and follow-up examination, and even reported persistence or recurrence of pain after the surgical correction [16]. Myofascial tightness is the most frequently proposed cause of back pain for AIS [17], but the effects from myofascial release techniques on pain release was not satisfactory [18]. Even though the individual has reached skeletal maturity and the curve stop progressing, the pain could persist and progress as daily lives goes on.

Bruyneel et al [9] reported that the AIS performed asymmetry bilateral loading pattern and increased loading at medial or lateral plantar surface as a function of the direction of scoliosis was observed. Therefore, it is reasonable to hypothesize that abnormal loading accumulated from daily walking might be one of the cause of back pain in AIS. To our knowledge, there is no study documented the characteristics of the walking pattern with measures that can provide loading information in AIS and its relation to scoliosis severity. This study investigates the walking patterns as a functional of scoliosis severity in AIS, which was measured by plantar CoP progression. We hypothesize that the plantar CoP progression pattern could change as a function of scoliosis severity. The research questions we would like to answer were: (1) Is the CoP progression pattern different between the AIS patients and normal subjects? (2) Is the CoP progression different among AIS groups with different scoliosis severity? And (3) Is the CoP progression of bilateral foot plantar surface symmetry among AIS groups?

## Materials and methods

### Subjects

**Fig 1:**
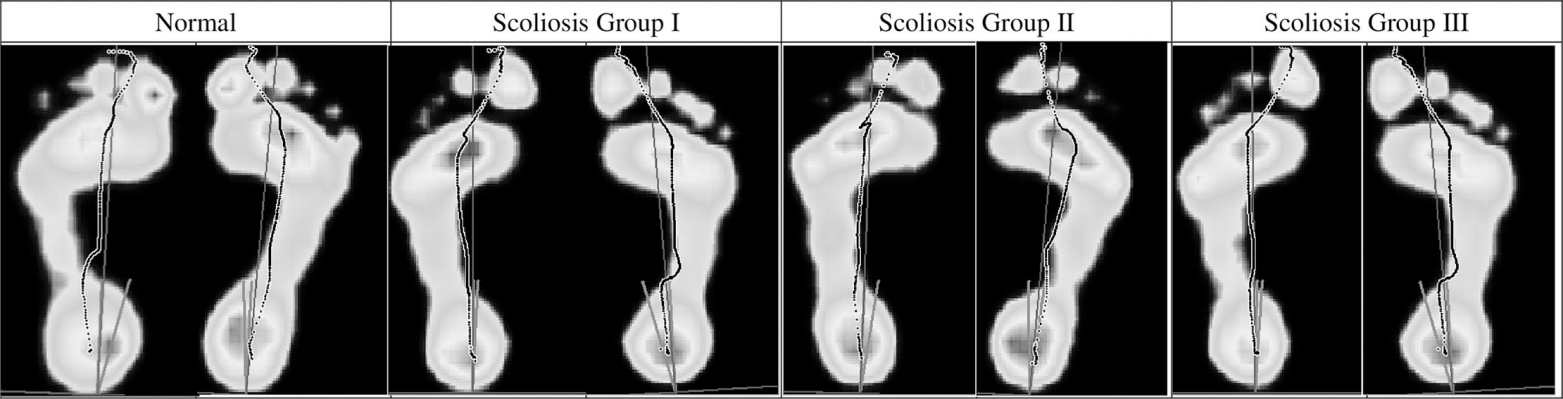
Foot prints outputted from the foot pressure measurement system. The foot prints showed the foot arches of subjects in various curve severity of idiopathic scoliosis groups and normal subjects and the figure was a single representative trial for a single typical subject in each group.

Thirty AIS patients were recruited intentionally to include even number of subjects of three IS severity based on the Cobb's angle in the major curve, which were mild IS group (Cobb's angle = 11°~25°, n = 10), moderate IS group (Cobb's angle = 26°~40°, n = 10), and severe IS group (Cobb's angle>40°, n = 10). Subjects from an orthopedic outpatient clinics and voluntary to participate in this study must fulfill the following inclusion criteria: aged between 12~18 and with the diagnosis of right-sided thoracolumbar scoliosis with the major curve at either the thoracic or lumbar portion of the spine for the first time, and no subjective complain of physical discomfort at the lower back region; and they were excluded when they were with any other known neurological or musculoskeletal abnormality or pathological conditions, with range of motion abnormality in the lower extremities, and/or with abnormal medial foot arch. Screening for the abnormal medial foot arch was made through visual inspection of the foot prints (Fig 1) which were extracted from the Footscan system while the subjects were maintaining an upright standing posture. The ankle-foot motion range was examined by an experienced occupational therapist who was blind to the purpose of this study and the results showed that the ankle joint flexibility in all the subjects were within normal range and no flat foot in any subject was observed. The diagnosis of AIS and severity of IS was determined by Cobb’s angle which was measured on a X-ray photography in frontal view by an orthopedic surgeon who was blind to our research purposes. No sagittal plane deformity, neither lordosis nor kyphosis, was noted in any AIS was found.

Thirty normal subjects who were comparable with AIS subjects in terms of age, body weight, body height and body mass index (BMI) were recruited from middle schools to be the normal group. The spine alignment in the normal subjects was screened by an occupational therapist using visual inspection and manual palpation of the back when the subjects performed the Adam’s Forward Bend test. Those who were observed with an asymmetry back alignment were excluded. The normal subjects were also excluded if their foot print shows any suspicion of abnormal height of the foot arch, which was examined with the same procedure as the AIS subjects.

All subjects with aged under 18 or under were entered into this study after their guardians signed the informed consent forms. The study was approved by the Chang Gung Medical Foundation Institutional Review Board. The parameters in all groups were compared with the design of case-control study with evidence of level III. Due to the limited AIS subjects, the main curve deformity in either the thoracic or lumbar portion may cause potential sources of bias.

### Experimental procedures

First, all subjects were asked to perform the Timed Up and Go test (TUG) as a measure of functional mobility, which requires both static and dynamic postural control. The given subject was instructed to perform a series of movements (rise from a chair without support, walk three meters, turn around, walk back to the chair and sit down) as fast as he/she could. The time required for completing these movements was measured by one of the authors by using a digital stop watch. Each subject has to perform TUG for three trials and the average time of seconds across three trials was used for statistical analysis. TUG has previously been suggested that scores of 4.98 to 5.69 seconds or less indicated normal mobility for adolescents in the age between 10 to 18 years of age [18].

Second, all subjects were asked to walk barefoot along a 5-meter walkway with a 0.5 meter pressure mat embedded in the middle. Three practice trials were allowed for each subject to be familiar with the setup and be confident about stepping on the mat with either the right or left foot. A trial was considered valid only when the subject stepped on the mat with only one foot (the right or the left) and the recording system captured a complete foot print. COP data of three valid trials for each foot were collected in each subject. The COP parameters were calculated first on each individual trial, then are averaged across three trials to get a single value for each subject and the single value of all subjects was used for statistical analysis.

The present study employed the foot pressure measurement system (Footscan, RSscan International Co., Belguim) included a 0.5-meter pressure mat (with 4096 active sensors) and a 3-D interface box in 500 Hz data acquisition rate, for recording the x-y coordinates of the CoP during the stance phase in level walking. Before data collection, the pressure mat was calibrated by default calibration software to reset the algorithm and determined the CoP coordinates in relation to the origin at the left-lower corner of the mat. This system has been used by several studies that measured human walking and is considered a well-accepted instrument for studying dynamic postural control during walking [18–19].

### Data processing

The CoP coordinates of each foot were exported and imported to a custom-written MATLAB program (MATLAB 7.0, MathWorks Inc.) for further data processing. The right and left foot data was processed and the results was reported separately to show the possible asymmetric influence of AIS on bilateral plantar CoP progression. The CoP data in a single trial was divided into three portions to show the CoP progression from the hindfoot, through the midfoot to the forefoot and toe regions. The CoP initiates from the heel upon heel contact and terminates between the big toe and the second toe upon toe-off. The progression of the CoP in the hindfoot region corresponds to the initial contact and loading-response sub-phase based on the Footscan gait 2^nd^ software and accounts for the initial 20% duration of the stance phase; that in the midfoot corresponds to the foot-flat sub-phase and accounts for the middle 60% during of the stance phase; that in the forefoot corresponds to push-off sub-phase and accounts for the terminal 20% duration of the stance phase. To avoid the interference of individual footprint on the above-mentioned approximation of the division of the three portions, the MATLAB program individually normalized each footprint into each of these sub-phases and forcing these percentages. The spatial parameters of the CoP trajectory under each portion of the plantar surface, i.e., the maximum medial-lateral displacement and anterior-posterior displacement, were calculated as resultants of movement control (inversion-eversion, dorsiflexion-plantarflexion) of the ankle-foot complex during walking. The zero displacement of the CoP was defined from the heel-2^nd^ toe foot axis. Foot length and foot width were considered significant factor interfering the CoP displacement within the foot but normalization of the CoP displacement to the foot length or width was not performed because that the CoP displacement of the three sub-phases has already been normalized to each individual foot dimensions.

The temporal parameters, i.e., the peak velocity and acceleration, were calculated representing the quality and amount of load accepted by and transferred upwardly from the plantar surface to the segments within the movement linkage of walking. All parameters of the right and left foot were calculated as reflections of the control of the four rockers (heel rocker, ankle rocker, forefoot rocker and toe rocker) within the ankle-foot complex that are responsible for the forward progression of the CoP and shock absorption when walking. They also are representations of inter-segment coordination of the apparatus within the locomotor system.

In summary, the spatial parameters calculated were the maximum CoP displacement in the medial-lateral (M-CoPD-ML) direction and the anterior-posterior direction (M-CoPD-AP). The temporal parameters included the timing (in terms of the percent of the stance phase) of peak CoP velocity (T-P-CoPV) and magnitude of peak CoP velocity (P-CoPV). In addition, Fig 1 shows a summary of the CoP trajectory of all subjects in each of the four groups.

### Statistical analysis

The differences among groups in terms of demographics, time of the TUG and CoP spatial-temporal parameters were analyzed by one-way analysis of variance (ANOVA), and the Sheffe’s post-hoc analysis was performed to analyze the differences between groups when a significant ANOVA analysis was found. Visual inspections of CoP roll-over trajectories were also performed to show the difference between all AIS and normal subjects, and the tendency of differing scoliosis severities in influencing CoP progression. All statistical analysis was done by using the SPSS 19.0 software package. The statistical significance level was set at *p* < .05.

## Results

### Demographic data

Table 1 shows, the subjects in four groups were comparable in age, body height, body weight and BMI (*p* > .05). All subjects were right-limb dominant. The mean BMI showed a tendency of decrement with IS severities. The Cobb's angles among three IS groups in the primary curves, which were at either the lumbar or the thoracic region, were significantly different (*p* < .05) and the major curve in all subjects were right sided; due to ethical and budget limitations, the Cobb’s angle was not measured in normal group. However, the spine deformity in sagittal plane (that is lordosis or kyphosis) was ruled out by surgeons’ examination on sagittal view X-ray. All the participants in this study have no stenosis. Nor any neurological compression symptom, such as numbness in the lower extremity and pain or discomfort in the lower back and lower limbs , was reported. Therefore, participants in mild scoliosis group were on the list of scoliosis progression monitoring, the participants in moderate scoliosis group were candidates for bracing, and participants in severe scoliosis group were candidates for surgery.

**Table 1:** Demographics data in four groups.

		Severity of IS					
Group	Normal (N = 30)	Mild IS (N = 10)	Moderate IS (N = 10)	Severe IS (N = 10)	*F*	*p*	η^2^
Age (y/o)	15.6±2.7	14.9±1.7	16.4±3.3	15.3±3.1	.56	.64	.03
Body Height (cm)	159.1±6.6	158.9±3.1	161.1±4.4	162.4±7.3	1.02	.38	.05
Body Weight (kg)	52.3±9.9	49.3±9.8	48.2±6.1	48.3±8.2	.84	.47	.04
BMI (kg/m^2^)	20.6±3.1	19.4±3.3	18.6±2.4	18.2±2.1	2.27	.09	.11
TUG (sec)	6.0±0.6^A^	6.8±1.5^B^	6.9±0.9^B^	6.5±0.8^A^	3.84	.01*	.171
Cobb's angle(°)	N/A	19.9±4.3^A^	31.8±4.2^B^	53.4±16.1^C^	29.35	.00*	.685

*p < .05

BMI: body mass index, TUG: Timed Up and Go test, IS: idiopathic scoliosis. Different alphabet superscript letters represent for significant difference in Scheffe post-hoc analysis (*p* < .05).

Results also showed that the four groups differed significantly in the time required to perform the TUG (*p* = .01). The normal group were able to finish the TUG within the shortest time duration (6.0±0.6 sec), while the moderate IS group needed the longest time duration (6.9±0.9 sec). The mild and moderate IS subjects has significantly higher TUG scores than the subject without IS (Table 1).

### Spatial parameters of plantar CoP

#### The right foot

Table 2 shows, the M-CoPD-ML within the midfoot (MF) region was significantly different among the groups (*p* = .01) and the post-hoc analysis showed that the CoP in normal and moderate IS groups shifted larger medial-laterally than that severe and mild IS groups did (*p* < .05). There were no significant differences in the hind-foot (HF) and fore-foot (FF) regions.

**Table 2:** Results of one-way ANOVA in CoP parameters between groups.

			Severity of IS				
Group	Normal (N = 30)	Mild IS (N = 10)	Moderate IS (N = 10)	Severe IS (N = 10)	*F*	*p*	η^2^
M-CoPD-ML (mm)							
R-HF	6.9±2.4	7.6±2.4	5.7±1.6	6.3±1.7	1.57	.20	.08
R-MF	8.2±2.8^A^	6.2±1.9^AB^	7.9±3.6^AB^	5.1±2.3^B^	3.89	.01*	.17
R-FF	13.3±5.6	15.0±6.9	14.3±9.2	15.0±5.2	.30	.82	.02
L-HF	5.5±2.1	7.7±5.8	5.8±2.1	6.0±2.1	1.36	.26	.07
L-MF	8.6±3.6	9.3±3.9	7.4±3.6	7.9±3.3	.57	.64	.03
L-FF	14.8±6.3	19.8±8.3	15.4±5.9	18.1±10.9	1.39	.25	.07
M-CoPD-AP (mm)							
R-HF	68.2±9.8	63.4±16.5	72.3±10.1	63.8±11.3	1.41	.25	.07
R-MF	95.2±10.1	94.7±13.5	89.4±9.3	97.2±12.7	.95	.42	.05
R-FF	45.1±5.5	50.8±8.0	51.2±9.8	47.9±6.7	2.87	.04^†^	.13
L-HF	69.9±11.2^A^	60.8±14.1^AB^	69.6±6.9^AB^	53.2±24.6^B^	4.10	.01*	.18
L-MF	93.3±11.3	99.5±13.9	94.9±7.9	102.5±18.8	1.58	.20	.07
L-FF	44.1±7.2	49.3±6.2	50.9±4.9	45.0±10.4	2.87	.05^†^	.13
P-CoPV (mm/sec)							
R-HF	1128.9±218.8^A^	816.7±182.8^B^	1037.6±289.7^AB^	798.9±163.0^B^	8.70	.00*	.32
R-MF	586.0±124.3	513.4±137.6	517.0±166.2	457.1±149.7	2.53	.07	.12
R-FF	810.4±114.0	822.0±155.0	854.4±226.4	796.7±139.7	.30	.83	.02
L-HF	1152.4±210.6^A^	876.2±389.7^AB^	1074.7±379.8^AB^	750.8±257.5^B^	6.09	.00*	.25
L-MF	616.7±152.8	522.9±144.8	536.2±86.1	484.2±134.8	2.92	.04^†^	.14
L-FF	799.0±119.6	758.0±173.9	778.3±136.9	693.7±219.6	1.24	.31	.06
T-P-CoPV (%)							
R-HF	10.3±2.3^AB^	10.1±2.8^AB^	8.3±1.5^A^	12.1±2.3^B^	4.56	.01*	.20
R-MF	51.1±7.9	54.0±10.4	51.1±9.6	55.3±9.5	.73	.54	.04
R-FF	93.8±1.3	93.0±1.5	91.9±2.3	93.8±2.4	2.94	.04^†^	.14
L-HF	11.3±3.3	11.1±2.1	9.8±2.3	11.0±1.9	.67	.57	.04
L-MF	53.0±9.0	55.0±7.4	57.5±6.6	57.1±7.9	1.09	.36	.06
L-FF	93.8±1.2	92.8±2.3	93.4±1.7	94.0±1.9	1.15	.33	.06

*p < .05

^†^*p* < .05 but without significant difference in post-hoc analysis

IS: idiopathic scoliosis, R: right foot, L: left foot, HF: hind foot, MF: midfoot, FF: forefoot, M-CoPD-ML: maximum CoP displacement in the medial-lateral direction, M-CoPD-AP: maximum CoP displacement in the anterior-posterior direction, P-CoPV: magnitude of peak CoP velocity, T-P-CoPV: percentage of the timing in stance phase occurred peak CoP velocity. Different alphabet superscript letters represent for significant difference in Scheffe’s post-hoc analysis (*p* < .05).

The M-CoPD-AP in the FF region was significantly different among the groups (*p* = .04) and there were no significant differences between groups found during post-hoc testing; only the tendency that the moderate IS group had larger anterior-posterior shift than the other groups. The M-CoPD-AP in the HF and MF regions were similar among the groups.

#### The left foot

The M-CoPD-ML in the HF, MF and FF regions did not show significant difference among the groups (Table 2). The M-CoPD-AP in the HF (*p* = .01) and FF (*p* = .05) regions were significantly different among groups; the post-hoc analysis showed that the normal and moderate IS groups had significantly larger M-CoPD-AP than the mild and severe IS groups (*p* < .05) had in HF region. However, there were no significant difference between groups found during host-hoc testing for M-CoPD-AP-FF in the left foot.

### Temporal parameters of plantar CoP

#### The right foot

Table 2 shows, the P-CoPV in the HF region was significantly different among the groups (*p* < .01), and the post-hoc analysis showed that the normal and moderate IS groups had a greater peak CoP velocity than the severe and mild IS groups did (*p* < .05); whereas, the P-CoPV in the MF and FF regions were similar among the groups.

The T-P-CoPV in the HF (*p* = .01) and FF (*p* = .04) regions were significantly different among the groups. The timing occurred peak CoP velocity in moderate IS group was significantly earlier than in the normal and severe IS group in HF area (*p* < .05). Post-hoc testing for the T-P-CoPV in the FF region for the right foot was not significantly different between groups. T-P-CoPV in moderate IS group tended to happen earlier than that in the other groups in the FF area (Table 2, *p* > .05).

#### The left foot

The P-CoPV in the HF (*p* = .00) and MF (*p* = .04) were significantly different among the groups, and the post-hoc testing showed that the normal and moderate IS groups had greater peak CoP velocity than the severe and mild IS groups did (*p* < .05) in the HF region; whereas, the post-hoc testing for P-CoPV in the FF region for the left foot were not significantly different between groups, indicating similar COP velocity in the forefoot region among the groups.

Finally, the T-P-CoPV did not show significantly different among the groups in all three portions of the left foot.

### Plantar CoP progression pattern

**Fig 2:**
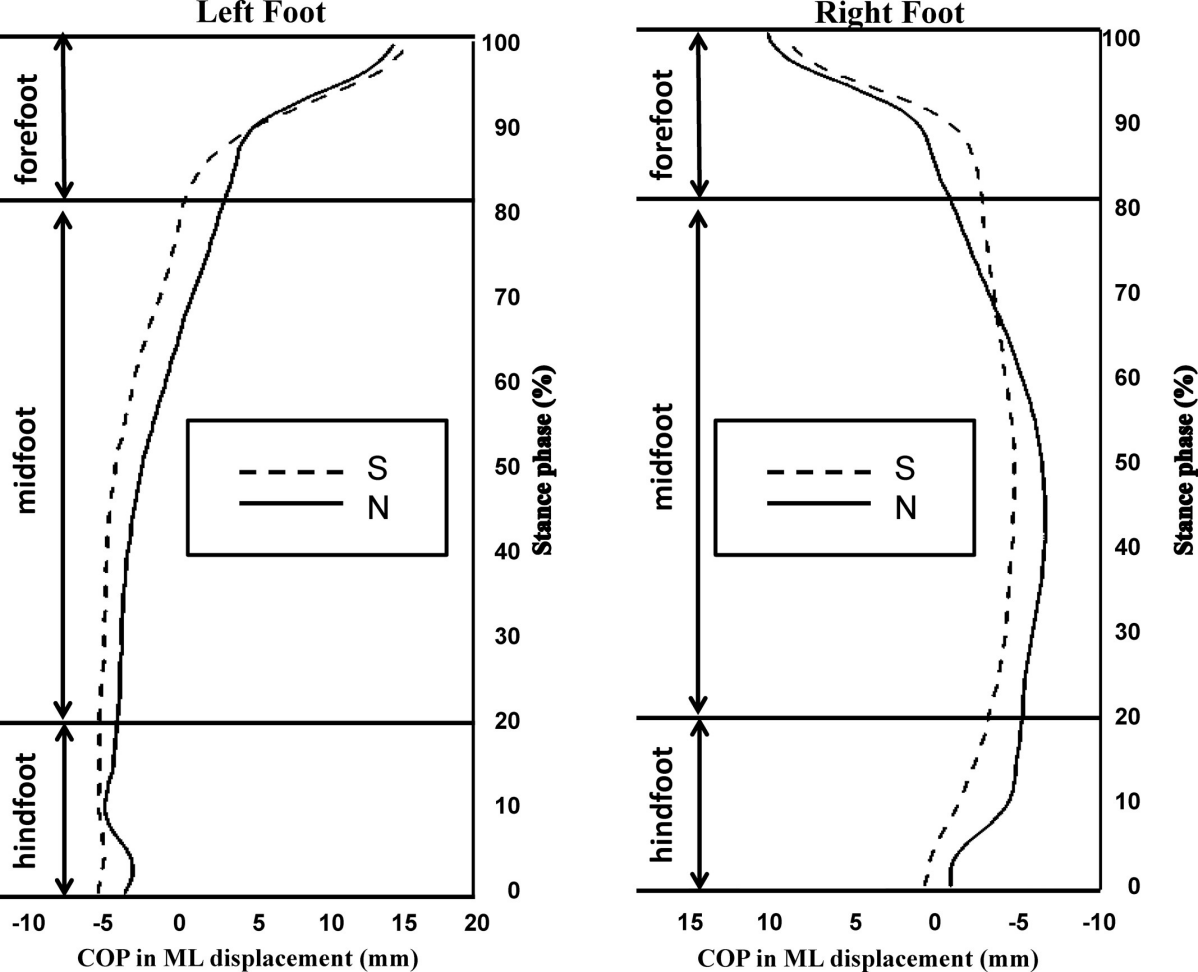
Center of pressure (CoP) progression in medial-lateral direction from hindfoot to forefoot region in all IS subjects (IS, dashed line) and normal subjects (N, solid line). The alphabetic symbols A~D in the figure indicated where the curves differed.

Fig 2 shows the average pediograms of both feet planter CoP progressed from the HF to the FF region between the normal group and all IS groups, and the asymmetry CoP trajectory was found in the IS group. Compared to the normal group, the CoP trajectory in the IS group left foot shifted laterally from the initial contact and travelled laterally all the way through the HF, MF and FF regions, but terminated at the similar location as the normal group. Results indicated that the foot of IS group contacted with the ground with more foot inversion than the normal group during level walking. On the other hand, the CoP trajectory in the IS group right foot shifted medially compared to the normal group and travelled medially through the HF region and most of the MF region. Then, the trajectory shifted laterally at the 65% of the stance phase and to the end of the stand phase.

**Fig 3:**
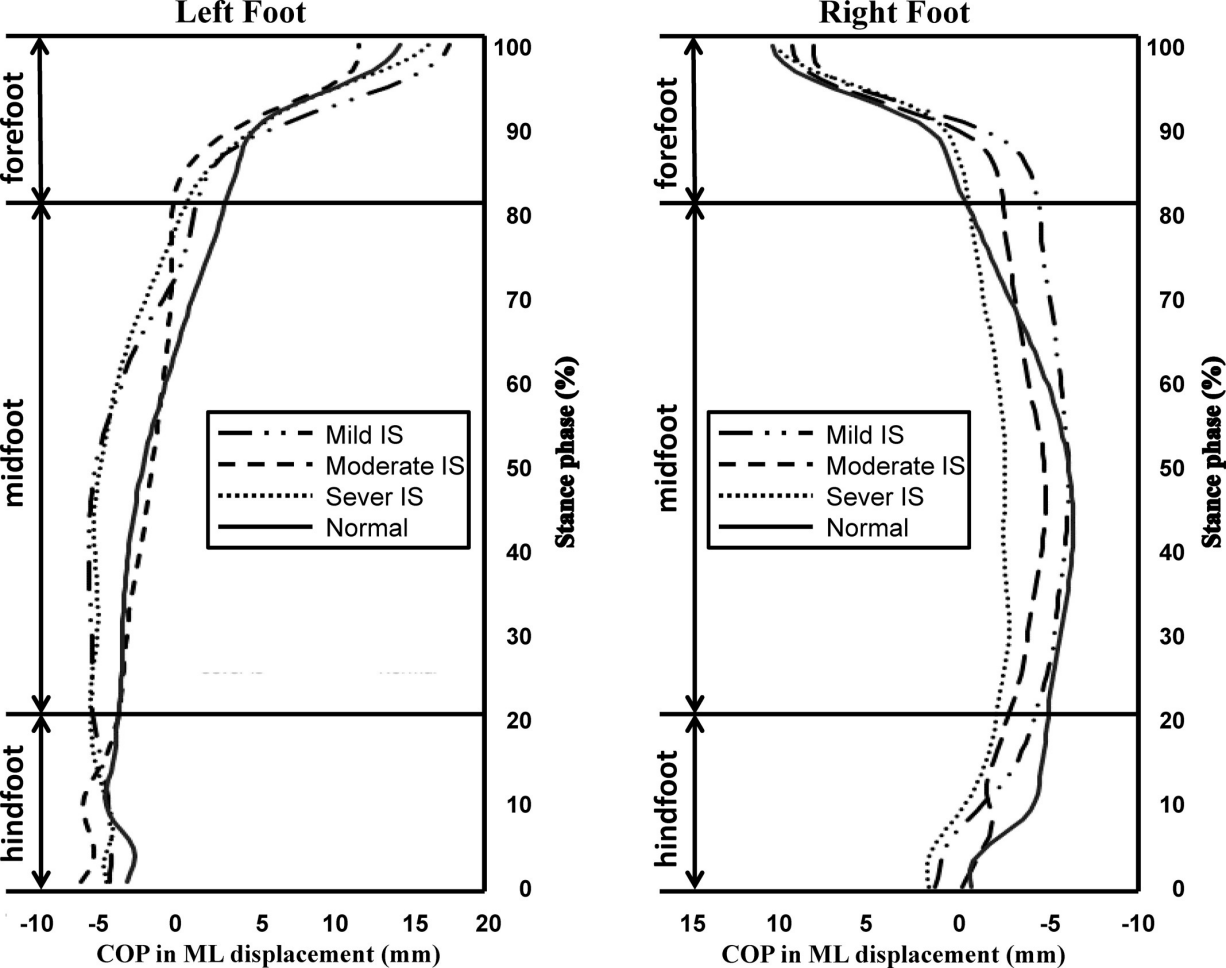
Center of pressure (CoP) progression in medial-lateral direction from hindfoot to forefoot region in adolescents with mild IS, moderate IS and severe IS groups. The alphabetic symbols A~F in the figure indicated where the curves differed.

Fig 3 shows the CoP progression patterns in three IS groups, and the asymmetry patterns were also found by the effects of the scoliosis severity. Results showed that the moderate IS group had most prominent deviation in level walking, the CoP initiated more laterally in both left and right heels, and then shifted medially during the MF region, especially in the left foot. This indicated that the moderate IS subjects contacted the ground with more foot inversion motion than others.

The mild and severe forms of IS was found with similar CoP trajectory patterns, which indicated the effects of IS on ankle-foot complex controlling CoP trajectory during level walking in mild and severe IS were similar. The severe IS group showed prominent CoP medial shift in right foot, which was consistent from the HF region and through the MF region to the end of the stance phase. The mild IS group performed a medial shift of the CoP in the right foot initial contact only, and it tended to shift laterally from the 10–88% of the stance phase.

## Discussion

It is well accepted that measures characterizing walking patterns are readily affected by age and anthropometric factors, and the contradictory findings regarding the walking parameters in AIS might be due to heterogeneity in the body statures of the subjects included in these studies [19]. Therefore, the body stature of subjects in this study were controlled by matched body weight and height across groups. However, a trend of decreasing BMI, which is calculated based on anthropometric data, as scoliosis severity increased was still noted. This finding indicated the potential association of musculoskeletal abnormality with the severity progression, which is in line with a previous study reported significant decrement in BMI in AIS subjects [19]. The role of the musculoskeletal abnormality in AIS development is supported [20], but the relation with scoliosis progression is still worthwhile for further exploration.

The walking speed measured during level walking through a 10-meter walk way is considered as a sensitive marker for identifying subjects with evident pathology such as Parkinsonism and subjects with mild cognitive impairment [21–24]. However, its sensitivity in identifying AIS and AIS with various scoliosis severity was discouraged by several recent studies [5,7]. Time recorded for completing TUG test, which is a standardized functional walking test, was proven to be a sensitive measure of walking functions in subjects with wide age ranges and various musculoskeletal or neurological conditions. [25–26]. Therefore, it was used in this study to explore its usability as a functional marker to identify AIS and AIS with various scoliosis severity.

Our results showed that the TUG score in AIS was much larger than that in normal subjects and the difference between the score in AIS and that in normal subjects was approaching the minimal detectable changes (MDC) (1.1seconds ~1.26 seconds) reported by previous studies measured TUG scores in children aged between 4 and 16 years. [18, 25–26]. Furthermore, mild and moderate IS groups was found spending significantly longer time than the severe IS group performing TUG but the difference in time to complete TUG between severe IS group and mild/moderate IS groups was far from the MDC reported in children [25–26]. In the view of neurology, chronic musculoskeletal disorders might associate with neuroplasticity [27–28] and, which subsequently changes the neuromechanism of motor control. Therefore, based on the finding of the difference in TUG score between the normal and the AIS, it is plausible to hypothesize the possible underlying changes of the neurological mechanism, which is responsible for walking speed control, in AIS. Our finding also indicates that scoliosis severity might not induce significantly enough differential changes of the neurological mechanism that controls functional walking in adolescents. This result support the use of TUG score as a functional marker to identify adolescents with IS.

Changes in biomechanical foundations due to scoliosis might be another contributing factor of the difference in functional walking performance, measured by TUG score, between groups. Want et al.’s [29] study indicated that the extent of pelvis axis rotation was associated with the Cobb’s angle on the primary curve in AIS and the magnitude of pelvis rotation in transverse plane could change the kinematic characteristics of walking pattern through the misaligned linkage of the pelvis, hip, knee and the angle-foot complex. The difference in TUG scores between AIS and normal groups and between severe and mild/moderate IS group might be due to accompanying different grades of pelvis rotation in AIS and AIS with various severity However, our results was based on small sample size in groups with various IS severity. a larger scale of study is needed to use TUG score as a functional marker to identify AIS with different severity.

Our finding of the CoP progression patterns of both feet during level walking between normal and AIS and between AIS with different severity of scoliosis supported previous studies suggesting the representativeness of CoP parameters as central mechanism of control of gait and posture [30–31]. The bilateral plantar CoP trajectories are reported as precise measurements of the biomechanical [11,30] and central mechanism [31] of ankle-foot complex during walking, and also reflected the loading pattern that the segments in the movement linkage bear. Our findings of changes in some of the CoP parameter between normal subjects and AIS and AIS with different severity indicated that the loading pattern and amount of loading that the segment within the movement linkage accepted tended to be changed due to the presence of IS in adolescents and the loading patterns also tended to be influenced by the severity of the scoliosis, which might partially explain the occurrence of back pain reported in AIS patients at their later life. The data also showed that the changes of bilateral plantar CoP measures in AIS and AIS with various severity was asymmetry, which might induce asymmetric back discomfort. Further studies should follow the relationship between walking pattern and subjective back pain of both side of the back reports with long-term scale studies for establishing a direct connection.

In this study, the plantar CoP trajectory of the right and left foot was measured and analyzed separately as it travelled from the hindfoot, through the midfoot to the forefoot region. Through the foot contacts to the ground, the foot accepts the loading and the load is transmitted upwardly to all the apparatus within the locomotor linkage system, which could assist the postural control adjustments. The position of CoP trajectory in relation to the foot axis (which is measured from the center of heel to the second toe) as one walks determines the action position of the ground reaction force in relation to the center of mass of the load transmitted upwardly. The velocity of CoP represents the speed of the foot contact with the ground and determines the amount of load transmitted upwardly from the plantar surface to the leg, thigh, and trunk. Our results showed position deviation of the CoP trajectory in AIS quantitatively (Table 2) and qualitatively (Figs 2 and 3), from that in aged-matched subjects without IS. Fig 2 summarizes the difference in CoP trajectory of both feet between normal and all AIS subjects (n = 30) without considering the difference in grades of scoliosis. The leftward (that is the convex side of the major scoliosis curve) shift of the CoP path under both feet at the hindfoot and midfoot portion was observed, and this finding indicates a less amount of heel inversion at initial contact and less pronation at foot flat. This might decrease the efficiency in contact loading absorption. This is an evidence supporting the possible association between IS and risk of ankle-foot injury and/or musculoskeletal discomfort of the lower limbs and the back. A possible contributing factor might be changes in the neuromuscular control of muscles around the ankle joint, as suggested by Chockalingam et al [9, 32] in response to the long-term development and or progression of peripheral musculoskeletal abnormality, such as development of scoliosis and scoliosis severity progression in this study. Furthermore, Table 2 and Fig 3 show that as scoliosis severity increased, the leftward shift of the CoP at initial contact tended to increase and the CoP travelled further medial to the heel-2^nd^ toe foot axis, which indicated less inversion-eversion transition during the stance phase as the scoliosis progressed. Heel eversion at initial contact was reported to be associated with higher heel skid velocity and premature medial loading to the foot, which might cause falls [33] and injuries to the ankle joint [34]. The results implied that scoliosis severity did change ankle-foot control mechanism during level walking for managing loading impact. This might also result in the reported lower back discomforted in subjects with severe scoliosis [35]. Another possible contributing factor for the motor performance deviation is neurological adaptation since the structural abnormalities of the ankle-foot complex were ruled out.

CoP displacement in the sagittal plane reflects the neural control of the plantar flexors for center of mass forward progression [36]. This denotes the sequential and qualitative control of four rockers within the ankle-foot complex. Table 2 shows that AIS subjects had less CoP anteroposterior displacement in the left hindfoot region and larger in the right forefoot region, which not only supports the asymmetric influence of IS on right and left foot motor performance [9] but also indicated that the changes of the heel/ankle rockers in feet becomes a functional modulation in AIS subjects, especially in the subjects with moderate and severe scoliosis.

Our results of CoP displacement of each foot during level walking between normal subjects and AIS and between AIS with various severity was not totally in line with the results of TUG scores (Table 2) but was a reasonable finding. Our argument is that TUG score is a result of bilateral lower limb and whole body motor coordination while CoP displacement reported in this study is the results of unilateral limb control, which tended to be influenced by IS asymmetrically and the ankle-foot mechanism of each foot. As reported by Chiu et al [13,18], the general walking speed is associated with plantar CoP velocity in healthy elderly, especially that in the midfoot area. Therefore, they argued that walking speed, which was cheaper and easier to measure than the plantar CoP velocity was, might be able to represent ankle-foot mechanism. Their study measured normal adults, who are considered symmetry organism with near but not perfect symmetric bilateral lower limb motor performance. Our results, showing asymmetric plantar CoP displacement of both feet, suggested that TUG though might be able to identify AIS but might not be as appropriate as plantar CoP displacement to identify IS effects on ankle-foot mechanism in each of the three portion of the foot.

Previous studies suggested the association of increased plantar CoP velocity during level walking at initial contact with increased risk of skipping falls in the elderly [11,37]. Our results showed that, on average, the peak CoP velocity of both feet at initial and mid stance in AIS tended to be slower than that in normal subjects of the same sub-phases, suggesting not only the changes of neurological mechanism for control of level walking but also the possible cautious ankle-foot control at these sub-phases. For daily living safety, AIS might have learned conservative gait control strategies by decreasing the instantaneously CoP velocity at initial contact [38]. On the other hand, the AIS accelerated their CoP at late stance sub-phase and the peak CoP velocity exceeded that in normal subjects, indicating increased risk of fall at a later stance in AIS as compared with the normal subjects. This study is the only study to date reporting increased peak CoP velocity at late stance in AIS. Our data suggested that the peak CoP velocity during level walking might be able to identify AIS from normal adolescents and risk of fall in AIS might be at the late stance phase when the toes are raising. Strategies for safety management during daily walking in AIS should be addressed.

In addition, our findings indicated that the scoliosis severity affected plantar CoP peak velocity during level walking, especially at the initial contact sub-phase which represents the control of heel and ankle rockers. The rocker system is a unique mechanism for the generation of forward momentum during walking. In this study, the average peak CoP velocity in the moderate IS group tended to be the fastest and that in severe IS group tended to be the slowest at initial contact and loading response sub-phases. It is reasonable to hypothesized that decreased CoP peak velocity might be an early sign of deterioration of IS severity in adolescents. In line with previous study [39], we vigorously suggest that moderate IS group, although was the least possible to be identified through changes of peak CoP velocity, might be at the highest risk of suffering from a fall injury due to lack of postural recovery mechanism once the walking stability was disturbed. The different central nervous mechanisms in controlling CoP progression patterns among scoliosis severity were noted. Nonetheless, the cause-effect relationship between IS deterioration and central nervous system impairment is yet to be conclusively established and the longitudinal follow-up study with more data are required.

Combining the finds in plantar CoP displacement and peak velocity, our finding indicated that plantar CoP displacement and peak velocity might be partially related. It was suggested that faster CoP velocity tended to cause increased CoP displacement. However, our finding failed to show the position relation between CoP displacement and CoP velocity. The reason might be the difference of CoP parameters measured and the method of CoP data processing. The plantar CoP displacement during level walking was measured in this study and it was normalized to the three sub-phases of the stance phase but not normalized to the foot dimensions. Furthermore, the peak plantar CoP velocity in each sub-phase of stance phase was used for analysis but not average CoP velocity of the whole stance phase. There is no study reported average plantar CoP velocity or examined its coupling with plantar CoP displacement in AIS. Therefore, it is not clear whether average plantar CoP velocity a better functional marker to identify AIS ro AIS with various severity.

The development of IS seemed not only changes the symmetric body structure and either the body functions into asymmetric ones. Our findings showed that the IS and the severity of IS was very likely to affect the CoP trajectory under both feet differently, which supported our hypothesis of asymmetric influence of IS on bilateral plantar CoP progression. Table 2, Figs 2 and 3 shows that the CoP progression under the right foot tended to be affected by IS and IS severity more than that under the left foot. The CoP displacement showed that the deviation of CoP trajectory was leftward as a function of IS and IS severity. Previous studies investigated the symmetry of gait in AIS with kinematic parameter is still debatable [36, 40–41]. Researchers have argued that the asymmetric spine curvature might associate with the abnormal somatosensory evoked potential in the posterior tibialis nerve (PTN) of the leg at the same side of convex of the curve [40]. The PTN is the primary nerve innervating the ankle muscles, which are responsible for controlling the ankle-foot motion in the frontal plane. Our data showed that only CoP displacement in the frontal plane within the IS subjects right foot was decreased, partially supported previously proposed impaired right leg PTN, which is on the same side of the convex of the major curve in AIS participated in our study. We further suggested that increasing scoliosis severity may further deteriorate the PTN function and affect the CoP displacement in the frontal plane subsequently. This is the first study that reports bilateral plantar CoP pattern quantitatively and qualitatively and found the asymmetry influence of IS and IS severity on CoP displacement and peak velocity. Our results indicated that the malalignment of the spine in the frontal plane might be associated with pelvis rotation in the transverse plane and affected bilateral limb motor control asymmetrically and might induce neuroplasticity and neurological mechanism changes.

## Conclusions

The plantar CoP derivatives during level walking was recorded in normal subjects and AIS subjects with various scoliosis severity. Results reaffirmed that (1) the AIS had different CoP trajectory patterns in terms of CoP trajectory position, CoP displacement, and CoP velocity, during level walking compared to the normal subjects, (2) the difference in CoP trajectory pattern among IS with different severity tended to be different, and (3) IS affected bilateral plantar CoP trajectory patterns asymmetrically. We hypothesized the possible contributing factors as changes in biomechanistic foundation of the linkage between spine-pelvis-thigh-leg-ankle-foot and possible neurological adaptation to the long-term development of IS. Our findings indicated that the AIS locomotors might have been subjected to abnormal burdens daily ever since when the IS first appeared and may related to the deterioration and accumulated musculoskeletal discomfort in the lower limb/trunk regions. Long-term, larger scale observation with sufficient number of participants (sample size of no less than 13 subjects in each group, calculated with statistical power of 0.8, alpha level = 0.05, and beta = 0,02) [42] using full spectrum of parameters crossing kinematics and kinetics is required to verify the relationship between changes in walking patterns and the occurrence of musculoskeletal discomfort in AIS. Rehabilitation professionals should advise AIS for proper interventions and prevention suggestions for their musculoskeletal discomfort, which might also be associated with scoliosis.
